# Supplementary material for: Intervention reducing malaria parasite load in vector mosquitoes: No impact on Plasmodium falciparum extrinsic incubation period and the survival of Anopheles gambiae
Source: PLoS Pathog. 2023 May 17;19(5):e1011084. doi: 10.1371/journal.ppat.1011084 (PMC10191285; doi:10.1371/journal.ppat.1011084)
Supplement: Attachment — Submitted filename: Summary Review of Guisso et al.docx [file ppat.1011084.s001.docx]

**Review of Guissou et al, PloS Pathogens**

This paper describes results of experiments designed to assess how variation in the gametocyte density of human blood infected with P. falciparum impacts the subsequent development time of parasites in mosquitoes (EIP) and their survival. The motivation is described as understanding the potential for ‘unexpected negative consequences’ of transmission-blocking interventions (TBIs), which would work by reducing but not completing blocking parasite transmission to mosquitoes. Strengths are the use of a real human malaria parasite system; including parasite isolates from natural infections and an outbred mosquito vector population. Other strengths are the use of a novel method for manipulating the density of parasites in the infectious blood meal while standardizing other components; and a novel non-invasive method for estimating when mosquitoes first become invasive. The resultant data would be of general interest and value to malaria-vector interactions.

However, there are some limitations and weaknesses that I recommend be addressed before this is published. In particular, engagement with the existing, quite large body of literature in this area is very superficial; and falls short in terms of contextualizing findings in the bigger picture. I have summarized key points below. Also more generally – there are quite a number of small grammar issues throughout (e.g LN 73 – should be ‘spraying’ not ‘sprayings’. LN 79 – should be “parasites” not ‘parasite’). I recommend the authors do a more thorough proof reading for the revised version.

Major

1) The abstract, author and intro focusses on ‘transmission blocking interventions’ (TBI)” as the

main motivation for the study. Without any specific reference to a TBI under development, and how the particular experiments carried out here mimic its mode of action, I find this quite speculative and abstract. In my opinion, it would be sufficient, and indeed more appropriate, to emphasize the fundamental research question asked by this study instead – do parasites in low density infections have an advantage by developing faster/having less impact on vector fitness and then reflecting on possible implications for control as a Discussion point; rather than framing the entire rationale for the study around TBIs. PloS Pathogens doesn’t require an applied angle, high quality fundamental vector-parasite interactions is sufficient. Rebalancing the abstract and intro to give a strong focus on these fundamental research questions would better reflect the results presented.

2) It would be useful to give more detail of the nature of results were given in the abstract. For example, results are summarized “*EIP and mosquito survival did not vary with parasite density, but different wit parasite isolates*” It would be useful to provide some details such as the number of parasite isolates compared, and the range in outcome variables (mean survival, EIP) between isolates to better convey the magnitude of these effects.

3) In the introduction, (pgh starting LN78) it would be good to give a more specific definition of how ‘transmission blocking’ is defined (if focus stays on this). Are you envisioning interventions that only block development of sporozoites, but not earlier stage gamete/ookinete development? Any TBI that blocked these entirely would not give any oocysts. . Clarify how ‘transmission blocking’ is defined in past work (e.g inability to develop sporozoite infections? No onward infection to a host etc?) In describing TBI in general, it would be good to give a clearer definition of the ways that ‘transmission blocking’ is defined and measured.

4) LN 99 – Authors raise the point that it could be problematic to use “% of sporozoite infected mosquitoes” as an indicator of exposure risk, as not all sporozoite-infected mosquitoes may be equally infectious (vary with sporozoite load). Examples are given of studies indicating that mosquitoes with higher sporozoite loads are infectious. Aren’t there also studies showing no association with sporozoite load and onward transmission? Could give a more balanced overview here. It would be useful to acknowledge that the number of sporozoites generated from one oocysts (several thousands) is well above the minimum needed to initiate one infection (~20-25 transmitted per bite).

5) LN 110- the hypothesis presented on parasite load influencing EIP seems weak and under-developed. A major gap is no description of a specific mechanism for why reducing parasite load would impact EIP; other than a vague reference to “competition”. I believe current evidence suggests that the length of the EIP is almost entirely determined by extrinsic factors like temperature. Can the authors point to any more specific evidence that the duration of sporogony is limited by mosquito resources? If not – this seems quite speculative. At very least – there should be a summary of what is currently known about the key factors that determine EIP length in mosquitoes (temperature, parasite genotype etc), and what if anything is known about whether mosquito resources play a role. Further, the explanation given in LN115 doesn’t make sense to me – if parasites are ‘*reducing investment in the production of transmission stages (gametocytes?)”,* why would this then impact the development time of the gamete in the mosquito?

When you return to this issue again in the Discussion (LN 240), again there is little mention of any concrete basis for predicting EIP to be limited by mosquito resources. To me it is not surprising that you found no impact of parasite density on EIP, as all the evidence I am aware of indicates this is almost entirely under the control of extrinsic factors. I think it would be more informative to acknowledge this point, rather than dedicating considerable space to arguing that the inability to find an association may have been due to a bias in the detection (as you do now). Unless you can provide a better justification of why such an association would be expected, I think your findings are entirely consistent with prediction. You mention one study that appeared to find this relationship (ref 34); but too little description of the findings is given to tell if it clearly showed EIP is limited by competition between parasites

6) In contrast to the unclear/uncertain justification of parasite load impacting EIP length, a much clearer rationale for hypothesizing that parasite load might impacts mosquito survival is given. Several studies have looked at this, with mixed results. The intro is somewhat unbalanced in cherry picking examples where mosquito survival appears was negatively associated with oocyst load, and not mentioning others where no association was found.

7) It would be useful to give some more details on experimental design – e.g how many mosquitoes used for each mosquito feed? How many “technical replicates” (separate feeds with different groups of mosquitoes) were done for each parasite density and isolate combination? Please add. Also, please provide more information on the biosecurity conditions under which mosquitoes were held (individually and in groups) to ensure no escape of sporozoite-infected mosquitoes. What steps were taken to ensure no accidental release of mosquitoes just about to become infectious?

8) Some aspects of the statistical analysis description were confusing. ‘Parasite isolate’ is described as being fit as a ‘random intercept’, but surely it had to be fit as fixed effect if you were doing significance testing (estimating a coefficient) for the impact of isolate?. What was the process of model selection? Backward elimination? Likelihood ratio tests, AIC, etc?

9) Fig 1a was confusing as the fitted black line and confidence interval shown give the impression that there was a positive association between gametocyte density and oocyst prevalence; but the text claims it wasn’t’? If there was no statistically significant association here, what does this black line represent? I had the same question about line on Figure 2a, which the text says is non-significant. Why show relationships on Figures if they are not statistically supportedt?

10) It would be useful to provide more info on the ‘cotton ball’ method of sporozoite detection in terms of its sensitivity and reliability. Could you summarize the ‘repeatability’/’reliability’ in terms of whether a cotton ball that tested positive one day would consistently do so in later tests? From the Discussion, it seems like a large proportion of mosquitoes who were ‘cotton ball negative’, did test positive for sporozoites via head/thorax qPCR.

11) In presenting a summary of how EIP varied between parasite isolates (LN 187) – you give single point values. Are these the mean/median EIP as estimate from the model? Or just central point from raw data? Clarify – and given some info on variability in these estimates (e.g confidence intervals if predicated from a model, max/min values if taken from raw data).

12) You found that infected mosquitoes had better survival than those that fed on infectious blood that did not develop an infection. This could be a result of a confounding factor; due to larger mosquitoes being both more likely to take big blood meals (and imbibe more parasites) and to live longer (body size is positively correlated with lifespan). Ideally you would have either measured blood meal size or wing length and incorporated these into analysis to control for this potential confounder. Currently you do not raise this when attempting to interpret this results in the Discussion (e.g starting LN 234). It would be useful to add this. Further in the Discussion (starting LN 267) there is an extended discussion of potential mechanisms for the enhanced survival of infected mosquitoes including mutualism or ‘cost of resistance’. As a confounding effect of body size is an equally (if not more?) likely explanation, this should be added.

13) LN 222 – reflects that positive association between gametocyte density and oocyst density found here was also reported in a previous study using a similar approach. However, there have been numerous studies that have tested for this association (although maybe not using the same protocol; e.g perhaps by exploiting naturally-occurring variation in gametocyte density in carriers). For completeness and context, it would be good tobriefly summarize how your findings (positive association) fits with the findings from this larger body of work.

14) LN231-232 – Here you assert that several mosquitoes that tested positive via qPCR on head/thorax could be false positives, as 99/201 of these did not produce a positive cotton ball. First as suggested above (pt 10)- this data on the proportion of qPCR positive that did not yield a positive cotton ball should be presented in the Results, Second – an alternative explanation for this is that the qPCR approach is just the most sensitive (better than oocyst dissection), and is much more likely to detect infection than a cotton ball sample. The latter seems much more likely to me – an infected mosquito may have 1000-10000s of sporozoites, whereas there may only be a few 10s of sporozoites expectorated onto a cotton ball in a given day. Surely it would be easier to get false negative with the cotton ball, than a false positive with the qPCR on head/thorax;?? Adding more reflection on the value/sensitivity of the cotton ball data; and how reliable it is for estimating parasite time courses, would be useful. Some focussed discussion on the strengths and limitations of this approach would be useful

15) The discussion of mosquito survival results lacks depth and doesn’t sufficiently connect with wider knowledge on this topic. In the paragraph starting LN 263 – it is stated ‘*one expectation is that higher parasite loads could affect longevity*..”. Please be more specific about the nature of the hypothesis you are making (e.g positive or negative, with explanation). In, LN 265 – please give refs to back up statement “*the question of the effect of plasmodium on mosquito survival being under debate for decades*”. Further in LN265, it is stated “the effect of dose-dependent effect *[note typo]* to be explored” – this is just not true. There are **lots** of studies that have investigated the impact of parasite load on mosquito survival, going back at least 20 years (e.g Martinez de la Puente etal 2018, Pollitt et al 2015, Dawes et al 2009, Ferguson et al 2003 and many others.) It is a notable omission not to make any reference to this wider body of work, and discuss how your findings fit within it. Other studies have found no association between parasite load and survival, similar to your results. Your current discussion of this finding is limited to statements of “*dose-dependent effects remaining to be explored”,* and “*more work being needed*”. A much better job of summarizing this previous work and situating your results within it is needed

16) Starting on LN 275, there is discussion of the mosquito survival results being consistent with potential trade-offs between survival and fecundity in infected mosquitoes. Several studies have looked at the impact of malaria infection on mosquito fecundity, including with simultaneous assessment of impacts on survival. Some of these could be incorporated to better support this hypothesis here.

Minor

-LN91 – I find this sentence a big convoluted and hard to follow. If a TBI works by reducing gametocyte density or infectiousness in general, then you may never get this situation of mosquitoes carrying high infection loads? You seem to be arguing that the average oocyst intensity in mosquitoes is independent of the TBI (e.g imperfect TBIs will have different impacts in settings where oocyst loads are ‘high’ versus ‘low’); which I think would be the case only if the TBI did not act on gametocytes, early stage parasites.

-LN 95- what is ‘transmission reducing activity”, how is this defined?

-I’m curious about the rationale for choice of dilutions. Why these particular gametocytes/ul? Is it known where the densities explored sit within the naturally-occuring range?
