# Supplementary material for: Intervention reducing malaria parasite load in vector mosquitoes: No impact on Plasmodium falciparum extrinsic incubation period and the survival of Anopheles gambiae
Source: PLoS Pathog. 2023 May 17;19(5):e1011084. doi: 10.1371/journal.ppat.1011084 (PMC10191285; doi:10.1371/journal.ppat.1011084)
Supplement: Attachment — Submitted filename: PLOS_Pathogens_Feb2023.docx [file ppat.1011084.s002.docx]

In this paper, the authors investigate whether decreasing the malaria parasite load within vector mosquitoes might have unintended consequences. Specifically, whether less heavily infected mosquitoes might live longer or if this might lead to parasites progressing through their life cycle more quickly (a shorter incubation period within the mosquitoes). The authors rightly point out that these factors must be properly considered before the impact of transmission-blocking interventions can be fully appreciated. A number of these tools are passing through the development pathways, which makes this work very timely. The variation in results seen for different parasite isolates is quite high, which makes it more difficult to extrapolate the implications of the findings more widely. Nevertheless, the use of natural isolates of *P. falciparum* is also one of the strengths of the work. The work is well presented, and I think it will be of interest to the malaria research community. I have a few comments and queries, however, that should be addressed before the article can be approved for publication. I commend the authors for this interesting work.

Regarding the relationship between gametocyte density and EIP (Figure 2), it is intriguing that parasite isolate B seems to behave quite differently to the other isolates. However, it is noticeable that fewer data points (n=13), were available for this isolate, is there a reason for this? Presumably this wasn’t planned. Furthermore, I’d be interested to know whether the upward slope observed for isolate B is significant in the regression model? This could be checked by including isolate as a fixed effect (I would say that a fixed effect is more suitable than a random effect for a variable with only three ’levels’, but I leave this choice to the authors).

The findings in Figure 3, regarding the relationship between parasite density and mosquito longevity are quite striking. The authors have given several possible explanations for this, and have outlined how future experiments could shed more light on this important issue. I have one concern about the interpretation of the results: perhaps the authors can reassure me about this. Specifically, whether the mosquitoes described as ‘exposed & uninfected’, might in fact be ‘exposed and not infected YET’. That is, if a mosquito dies before parasites develop sufficiently in order to be detected (it might be useful to restate in the caption how infection is being measured here, I think it’s sporozoite prevalence by PCR, but I’m not certain), this could confound the results. In other words, if a mosquito needs a long lifetime in order to be measured as infected, then infected mosquitoes may appear to be more long-lived. I am slightly reassured that it appears that most mosquitoes live for two weeks or more, meaning that few mosquitoes should die during the extrinsic incubation period. I would be interested to know if the authors can reassure me on this point. If not, it should be listed as a limitation of the work.

Relating to the previous question on Figure 3a, it appears that almost no mosquitoes die in the first 9 days. Have I interpreted that correctly? Or have those mosquitoes been removed from the results (e.g. if they don’t live long enough for their infection status to become measurable)?

Other comments:

Page 2 line 42: I would change this to ‘Our results here do not identify…’, but I leave this choice to the authors

Page 4 line 79: I would suggest either ‘targeting parasites within the mosquito’, or ‘targeting the parasite within the mosquito’

Page 6 line 146: I would suggest ‘… at 7 days post blood meal’
