# Supplementary material for: Intervention reducing malaria parasite load in vector mosquitoes: No impact on Plasmodium falciparum extrinsic incubation period and the survival of Anopheles gambiae
Source: PLoS Pathog. 2023 May 17;19(5):e1011084. doi: 10.1371/journal.ppat.1011084 (PMC10191285; doi:10.1371/journal.ppat.1011084)
Supplement: Attachment — Submitted filename: Reponse to editor and reviewers comments 12april23.docx [file ppat.1011084.s003.docx]

***Dear Kenneth D Vernick, Ronald Swanstrom, Kasturi Haldar and Michael Malim***

***Thank you for reviewing our manuscript entitled* " Intervention reducing malaria parasite load in vector mosquitoes: no impact on *Plasmodium falciparum* extrinsic incubation period and the survival of *Anopheles gambiae* "*.***

***We greatly appreciate the input we received from you and the reviewers. Especially, we acknowledge the great work of the editor to gather and combine the comments of the five reviewers in the summary document. Below, we answered to each specific points of this summary document. We hope that you will find this revised version acceptable for publication in* Plos Pathogens*.***

***Sincerely,***

***Edwige Guissou on behalf of all co-authors.***

**Review of Guissou et al, PloS Pathogens**

This paper describes results of experiments designed to assess how variation in the gametocyte density of human blood infected with P. falciparum impacts the subsequent development time of parasites in mosquitoes (EIP) and their survival. The motivation is described as understanding the potential for ‘unexpected negative consequences’ of transmission-blocking interventions (TBIs), which would work by reducing but not completing blocking parasite transmission to mosquitoes. Strengths are the use of a real human malaria parasite system; including parasite isolates from natural infections and an outbred mosquito vector population. Other strengths are the use of a novel method for manipulating the density of parasites in the infectious blood meal while standardizing other components; and a novel non-invasive method for estimating when mosquitoes first become invasive. The resultant data would be of general interest and value to malaria-vector interactions.

However, there are some limitations and weaknesses that I recommend be addressed before this is published. In particular, engagement with the existing, quite large body of literature in this area is very superficial; and falls short in terms of contextualizing findings in the bigger picture. I have summarized key points below. Also more generally – there are quite a number of small grammar issues throughout (e.g LN 73 – should be ‘spraying’ not ‘sprayings’. LN 79 – should be “parasites” not ‘parasite’). I recommend the authors do a more thorough proof reading for the revised version.

***We agree with the comment that the contextualization of the study and the state of the art were superficial and we now gave a much more complete picture with a more theoretical point of view in the introduction (lines 106 to 147 in the version with track changes), but also the discussion (from line 339 to 373).***

***We corrected the grammatical errors now in line 79 and line 85. In addition, the revised manuscript has been proofread by a native English speaker*.**

1) The abstract, author and intro focusses on ‘transmission blocking interventions’ (TBI)” as the main motivation for the study. Without any specific reference to a TBI under development, and how the particular experiments carried out here mimic its mode of action, I find this quite speculative and abstract. In my opinion, it would be sufficient, and indeed more appropriate, to emphasize the fundamental research question asked by this study instead – do parasites in low density infections have an advantage by developing faster/having less impact on vector fitness and then reflecting on possible implications for control as a Discussion point; rather than framing the entire rationale for the study around TBIs. PloS Pathogens doesn’t require an applied angle, high quality fundamental vector-parasite interactions is sufficient. Rebalancing the abstract and intro to give a strong focus on these fundamental research questions would better reflect the results presented.

***We thank the referee for this comment and agree that the focus on TBI was too strong and occupying the entire introduction. We reduced it drastically and emphasized the theoretical and evolutionary context. Although reduced, we still kept the applied angle because we are quite convinced that this may attract both the community of readers interested in malaria control and their consequences as well as readers interested in the evolutionary ecology of host-parasites interactions. The majority of the reviewers also appeared to find the TBI approach original and timely. We have better explained how TBIs work and how their efficacy is measured (see below), which should make the rationale more convincing.***

***We hope that, by improving the balance between fundamental and applied literature in the introduction we provided a more convincing rationale.***

***.***

2) It would be useful to give more detail of the nature of results were given in the abstract. For example, results are summarized “*EIP and mosquito survival did not vary with parasite density, but different wit parasite isolates*” It would be useful to provide some details such as the number of parasite isolates compared, and the range in outcome variables (mean survival, EIP) between isolates to better convey the magnitude of these effects.

***We have made the corresponding modifications in the manuscript in the abstract and main text (see lines 39-40, 44 to 46, 246-247, 260-261).***

3) In the introduction, (pgh starting LN78) it would be good to give a more specific definition of how ‘transmission blocking’ is defined (if focus stays on this). Are you envisioning interventions that only block development of sporozoites, but not earlier stage gamete/ookinete development? Any TBI that blocked these entirely would not give any oocysts. . Clarify how ‘transmission blocking’ is defined in past work (e.g inability to develop sporozoite infections? No onward infection to a host etc?) In describing TBI in general, it would be good to give a clearer definition of the ways that ‘transmission blocking’ is defined and measured.

***To make the rationale more comprehensive, we explained better how TBIs work (lines 86-92) and how their efficacy is measured (lines 92-95) and the density dependent efficacy (lines 95-99). We fully agree that the previous version was confusing: when saying that TBI efficacy depends on the density of oocyst stage it suggests that they target late stage of infection (oocyst and sporozoite). This is not the case: they target early stages (gametes to ookinetes) and the efficacy is measured by comparing oocyst intensities between groups of mosquitoes exposed to an infectious blood meal with versus without the blocking agent. Therefore, oocyst nb is the measured outcome and is used as a proxy of the subsequent infectivity to next vertebrate host.***

***When we say that efficacy depends on density this is because when the infection load is high in the control group, the efficacy of TBI at clearing the infection in the test group is reduced (in other words they are less efficient at blocking all 50 ookinetes than 3 ookinetes). We hope that this explantion makes the introduction clearer***

4) LN 99 – Authors raise the point that it could be problematic to use “% of sporozoite infected mosquitoes” as an indicator of exposure risk, as not all sporozoite-infected mosquitoes may be equally infectious (vary with sporozoite load). Examples are given of studies indicating that mosquitoes with higher sporozoite loads are infectious. Aren’t there also studies showing no association with sporozoite load and onward transmission? Could give a more balanced overview here. It would be useful to acknowledge that the number of sporozoites generated from one oocysts (several thousands) is well above the minimum needed to initiate one infection (~20-25 transmitted per bite).

***This is an interesting point, but we removed this sentence, not directly related to our study, when reducing the focus on TBI.***

5) LN 110- the hypothesis presented on parasite load influencing EIP seems weak and under-developed. A major gap is no description of a specific mechanism for why reducing parasite load would impact EIP; other than a vague reference to “competition”. I believe current evidence suggests that the length of the EIP is almost entirely determined by extrinsic factors like temperature. Can the authors point to any more specific evidence that the duration of sporogony is limited by mosquito resources? If not – this seems quite speculative. At very least – there should be a summary of what is currently known about the key factors that determine EIP length in mosquitoes (temperature, parasite genotype etc), and what if anything is known about whether mosquito resources play a role. Further, the explanation given in LN115 doesn’t make sense to me – if parasites are ‘*reducing investment in the production of transmission stages (gametocytes?)”,* why would this then impact the development time of the gamete in the mosquito?

When you return to this issue again in the Discussion (LN 240), again there is little mention of any concrete basis for predicting EIP to be limited by mosquito resources. To me it is not surprising that you found no impact of parasite density on EIP, as all the evidence I am aware of indicates this is almost entirely under the control of extrinsic factors. I think it would be more informative to acknowledge this point, rather than dedicating considerable space to arguing that the inability to find an association may have been due to a bias in the detection (as you do now). Unless you can provide a better justification of why such an association would be expected, I think your findings are entirely consistent with prediction. You mention one study that appeared to find this relationship (ref 34); but too little description of the findings is given to tell if it clearly showed EIP is limited by competition between parasites

***We provided a summary of the current knowledge on the factors affecting EIP (environmental conditions and genetics). We hope that the more complete review of literature now presented in the introduction is more convincing. We cite studies that show the effect of temperature on EIP but also 3 references that show an effect of nutritional resources. We think that an effect of nutritional resources is a good reason to hypothesize that competition may occur for these resources and that density dependent effect may occur. (lines 118 to line 136)***

***In this paragraph, when talking about transmission stages, we meant transmission from mosquito to human, e.i. sporozoites. We agree that transmission stages usually refer to gametocytes and we clearly cite “sporozoites” to avoid confusion (line 120).***

6) In contrast to the unclear/uncertain justification of parasite load impacting EIP length, a much clearer rationale for hypothesizing that parasite load might impacts mosquito survival is given. Several studies have looked at this, with mixed results. The intro is somewhat unbalanced in cherry picking examples where mosquito survival appears was negatively associated with oocyst load, and not mentioning others where no association was found.

***A more complete review of literature is now cited, giving examples of contrasting results and showing the diversity of results (lines 139-146)***

7) It would be useful to give some more details on experimental design – e.g how many mosquitoes used for each mosquito feed? How many “technical replicates” (separate feeds with different groups of mosquitoes) were done for each parasite density and isolate combination? Please add. Also, please provide more information on the biosecurity conditions under which mosquitoes were held (individually and in groups) to ensure no escape of sporozoite-infected mosquitoes. What steps were taken to ensure no accidental release of mosquitoes just about to become infectious?

***We added some clarifications in the manuscript (see lines 439 to 450)***

8) Some aspects of the statistical analysis description were confusing. ‘Parasite isolate’ is described as being fit as a ‘random intercept’, but surely it had to be fit as fixed effect if you were doing significance testing (estimating a coefficient) for the impact of isolate?. What was the process of model selection? Backward elimination? Likelihood ratio tests, AIC, etc?

***We agree that some aspects of the statistical analysis were confusing in the previous version. We have clarified all this in the revised version. The primary objective of this study was to explore the effect of parasite density on EIP. Two proxies were used for parasite density: gametocyte density (Fig 2a) and sporozoite quantity (Fig 2b). The experiment was performed three times each using a different parasite isolate (isolates A-C). Because these isolates came from different donors (and each with a different initial gametocytemia), there was non-independence / pseudoreplication among isolates, which must be controlled carefully by setting isolates as a random effect in the mixed models. Therefore, in our analyses, EIP was first analysed using two LMMs, the first specifying gametocyte density as a fixed and a random slope factor and parasite isolate as a random intercept (Fig 2a), the second specifying sporozoite load in mosquito head/thorax as a fixed and a random slope factor and parasite isolate as a random intercept (fig 2b). In these models, the statistical significance of the fixed effects (i.e. gametocytemia in fig2a and sporozoite load in fig 2b) was evaluated using the “Anova” function of the “car” package. This function calculates type-II or type-III analysis-of-variance tables for a wealth of different model objects including glmer objects from the lme4 package, and can be assimilated to a step wise deletion approach using LRT tests for model comparisons.***

***Second, we also investigated whether EIP varied among isolates, which might suggest the existence of genetic variability underlying EIP. Accordingly, significance of the isolate variable was reported at lines 246-247 (Figure 2c, effect of isolates on EIP). For this analysis, the variable “isolate” was, as outlined by referee, considered as a fixed parameter in a Cox model. Since the outcome variable here is the time when sporozoites are first detected in cottons from individual mosquito, we used the statistical approach specifically developed for investigating the time a specified event takes to happen, that is, Cox proportional hazard models (most often used to analyze time to death or time to recovery). Therefore we performed such a cox model setting isolate as the only fixed effect to test for statistical difference in sporozoites appearance time (or EIP by extension) among parasite isolates (Figure 3c).***

***We have adopted exactly the same approach to analyze (i) the effect of density (gametocytemia or sporozoite load) on mosquito longevity i.e. 2 mixed models specifying isolate as random effect: figure 3c and 3d) and (ii) the effect of parasite isolate on mosquito survival (a cox model with isolate as a fixed effect, fig3e).***

***Concretely, we now indicate in the main text at lines 513 and 521 that parasite isolate was considered as a fixed effect in the “sporozoites appearance time (or EIP by extension) Cox model” and in “the mosquito survival Cox model”.***

***Finally, we have prepared all the analysis R scripts/codes and the accompanying dataset to share them. These data and code files are available on a dataverse repository:***

<https://dataverse.ird.fr/privateurl.xhtml?token=dacc1813-875e-4052-a3d5-c737dbff9ca5>

***These scripts are arranged according to figures such that it will be easy to connect the model structures for each of the different panels of all figures.***

9) Fig 1a was confusing as the fitted black line and confidence interval shown give the impression that there was a positive association between gametocyte density and oocyst prevalence; but the text claims it wasn’t’? If there was no statistically significant association here, what does this black line represent? I had the same question about line on Figure 2a, which the text says is non-significant. Why show relationships on Figures if they are not statistically supportedt?

***We thank the referee for this comment, we fully agree with it. We removed the relationship when not significant, so not only for 1a and 2a but also 3b, 3c and 3d.***

10) It would be useful to provide more info on the ‘cotton ball’ method of sporozoite detection in terms of its sensitivity and reliability. Could you summarize the ‘repeatability’/’reliability’ in terms of whether a cotton ball that tested positive one day would consistently do so in later tests? From the Discussion, it seems like a large proportion of mosquitoes who were ‘cotton ball negative’, did test positive for sporozoites via head/thorax qPCR.

***We acknowledge the pertinence of the question***.

***An important proportion of the mosquitoes that were found positive in the head-thorax never produced positive cotton (first line of the table below). This is clearly stated and discussed in the manuscript (“ Of the 201 females with infected head/thorax, 102 (50.7%) generated at least one cotton ball containing detectable traces of parasite DNA” lines 235-237).***

***Moreover, among the mosquitoes that already produced a positive cotton ball, the majority of cotton remains negative (third line of the table below). This is now clearly stated in the result section (lines 240-241) and in the discussion (lines 324-325). This is largely discussed in the paper that described this non destructive method (Guissou et al. scientific reports, 2021). It can be due to sensibility issues but also due to the fact that mosquitoes do not sugar feed every day (supplementary table 6 of Guissou et al. 2021). The implication of this observation is a possible overestimation of the EIP (the first detection of plasmodium DNA in a cotton may happen latter than the actual presence of sporozoites in salivary glands) (lines 329-330).***

***A possible issue due to sensibility could have happened in our study if highly infected mosquitoes had showed shorter EIP: we could not discriminate between better detection due to higher DNA load and actual quicker EIP. The fact that EIP was not significantly affected by parasite density allows us to exclude the potential false result due to sensibility. This is discussed (lines 331-333).***

**Table: Proportion of mosquitoes producing at least one positive cotton over the collection period and proportion of positive cottons both over the collection period and after the first positive detection (“post-EIP”) for each parasite isolate.**

|  | **collection period** | ***Isolate A*** | ***Isolate B*** | ***Isolate C*** |  |
| --- | --- | --- | --- | --- | --- |
| **Proportion of infected mosquitoes generating at least one positive cotton** | **8-56 dpbm** | **0.58 (26/45)** | **0.19 (13/67)** | **0.71 (63/89)** | |
| **Proportion of positive cottons** | **8-56 dpbm** | **0.12 (238/2001)** | **0.03 (26/936)** | **0.21 (632/3054)** | |
| **Proportion of positive cottons** | **post EIP** | **0.26 (238/916)** | **0.23 (26/115)** | **0.39 (632/1619)** | |

11) In presenting a summary of how EIP varied between parasite isolates (LN 187) – you give single point values. Are these the mean/median EIP as estimate from the model? Or just central point from raw data? Clarify – and given some info on variability in these estimates (e.g confidence intervals if predicated from a model, max/min values if taken from raw data).

***The presented values are median EIP that were estimated by the model and we added the confidence intervals. This is now clarified and completed in the manuscript (abstract and lines 246 to 247).***

12) You found that infected mosquitoes had better survival than those that fed on infectious blood that did not develop an infection. This could be a result of a confounding factor; due to larger mosquitoes being both more likely to take big blood meals (and imbibe more parasites) and to live longer (body size is positively correlated with lifespan). Ideally you would have either measured blood meal size or wing length and incorporated these into analysis to control for this potential confounder. Currently you do not raise this when attempting to interpret this results in the Discussion (e.g starting LN 234). It would be useful to add this. Further in the Discussion (starting LN 267) there is an extended discussion of potential mechanisms for the enhanced survival of infected mosquitoes including mutualism or ‘cost of resistance’. As a confounding effect of body size is an equally (if not more?) likely explanation, this should be added.

***We thank the referees for raising this possible explanation that we added in the manuscript (see lines 351 to 354).***

13) LN 222 – reflects that positive association between gametocyte density and oocyst density found here was also reported in a previous study using a similar approach. However, there have been numerous studies that have tested for this association (although maybe not using the same protocol; e.g perhaps by exploiting naturally-occurring variation in gametocyte density in carriers). For completeness and context, it would be good tobriefly summarize how your findings (positive association) fits with the findings from this larger body of work.

***This is indeed interesting to mention other studies on the relationship between gametocyte density and infection load. We now cite an important meta-analysis on this subject (line 287-289)***

14) LN231-232 – Here you assert that several mosquitoes that tested positive via qPCR on head/thorax could be false positives, as 99/201 of these did not produce a positive cotton ball. First as suggested above (pt 10)- this data on the proportion of qPCR positive that did not yield a positive cotton ball should be presented in the Results, Second – an alternative explanation for this is that the qPCR approach is just the most sensitive (better than oocyst dissection), and is much more likely to detect infection than a cotton ball sample. The latter seems much more likely to me – an infected mosquito may have 1000-10000s of sporozoites, whereas there may only be a few 10s of sporozoites expectorated onto a cotton ball in a given day. Surely it would be easier to get false negative with the cotton ball, than a false positive with the qPCR on head/thorax;?? Adding more reflection on the value/sensitivity of the cotton ball data; and how reliable it is for estimating parasite time courses, would be useful. Some focussed discussion on the strengths and limitations of this approach would be useful

***We mentioned in the result section the proportion of mosquitoes positive by qPCR that did not produce a positive cotton ball (lines 236-239 : Of the 201 females with infected head/thorax, 102 (50.7%) generated at least one cotton ball containing detectable traces of parasite DNA. The infected females that did not produce any positive cottons over their lifespan were excluded from the analysis because no EIP values can be derived from these samples).***

***In the first version of the discussion, we suggested that false positive of qPCR for head and thorax because we obtained a higher prevalence with this method compared to oocyst detection, which is usually quite sensitive. It is indeed likely that the higher prevalence in head thorax compared to cotton balls is probably a combination of 2 effects (false positive among head/thorax+ low sensitivity of cotton test)***

***Therefore,*** ***we agree with the comment on the need to better explain the sensitivity matter, which we now do, lines 323-328.***

15) The discussion of mosquito survival results lacks depth and doesn’t sufficiently connect with wider knowledge on this topic. In the paragraph starting LN 263 – it is stated ‘*one expectation is that higher parasite loads could affect longevity*..”. Please be more specific about the nature of the hypothesis you are making (e.g positive or negative, with explanation). In, LN 265 – please give refs to back up statement “*the question of the effect of plasmodium on mosquito survival being under debate for decades*”. Further in LN265, it is stated “the effect of dose-dependent effect *[note typo]* to be explored” – this is just not true. There are **lots** of studies that have investigated the impact of parasite load on mosquito survival, going back at least 20 years (e.g Martinez de la Puente etal 2018, Pollitt et al 2015, Dawes et al 2009, Ferguson et al 2003 and many others.) It is a notable omission not to make any reference to this wider body of work, and discuss how your findings fit within it. Other studies have found no association between parasite load and survival, similar to your results. Your current discussion of this finding is limited to statements of “*dose-dependent effects remaining to be explored”,* and “*more work being needed*”. A much better job of summarizing this previous work and situating your results within it is needed

***We agree that our contextualization with previous work was way too brief and stated our hypothesis with shortcuts. With a better description of the state of the art in the introduction, and reminders in the discussion and better link with our hypothesis, we hope that the context of our study is much clearer (lines 342-346). Importantly, we now make the difference between model vector parasite systems and human malaria vector parasites species combinations, which can show substantial differences.***

16) Starting on LN 275, there is discussion of the mosquito survival results being consistent with potential trade-offs between survival and fecundity in infected mosquitoes. Several studies have looked at the impact of malaria infection on mosquito fecundity, including with simultaneous assessment of impacts on survival. Some of these could be incorporated to better support this hypothesis here.

***We now cite a panel of studies that recorded survival and fecundity in regard to infection in malaria vectors (lines 367-369). It remains difficult to draw a general conclusion from the conflicting results and we discuss the added value of the non-destructive method to follow infection which may help on this question as well.***

Minor

-LN91 – I find this sentence a big convoluted and hard to follow. If a TBI works by reducing gametocyte density or infectiousness in general, then you may never get this situation of mosquitoes carrying high infection loads? You seem to be arguing that the average oocyst intensity in mosquitoes is independent of the TBI (e.g imperfect TBIs will have different impacts in settings where oocyst loads are ‘high’ versus ‘low’); which I think would be the case only if the TBI did not act on gametocytes, early stage parasites.

***We now explained how the efficacy of TBI is measured (lines 92-94)and hope that the relation between oocyst load and efficacy is now clear (lines 95-99). TBI always act so far before oocyst stage, we then measure the efficacy of the intervention by comparing the number of oocysts between groups of mosquitoes exposed or not to the blocking agent (in the control group the mean nb of oocysts is not affected, it is in the test group). It is now described that when the mean oocyst nb is high in the control group, the mean nb of oocyst is affected by TBI in the test group, but the decrease of infection prevalence (the efficacy of the intervention) is limited***.

-LN 95- what is ‘transmission reducing activity”, how is this defined?

***We removed this part, but for your information, TRA is the impact of the intervention on oocyst load (compared to the control group of mosquitoes) which is constant whatever the oocyst load in the control group. In contrast the TBA is the impact of the intervention on oocyst prevalence (compared to the control group of mosquitoes) which is negatively related to the oocyst load in the control group* (churcher et al 2012)*.***

-I’m curious about the rationale for choice of dilutions. Why these particular gametocytes/ul? Is it known where the densities explored sit within the naturally-occuring range?

***We selected high gametocyte loads among naturally infected volunteers, so that with dilution, we reproduce a range of densities that may mimic natural highest infection loads and the ones reduced by TBI (this is now precised line 280-282). Cf figure in supplementary data from Bompard et al. 2020 showing gametocyte densities in two villages (red and blue) in the vincinity of Bobo Dioulasso.***

***And the range of oocyst load also correspond to naturally infected mosquitoes (Bompard et al. 2020) :***

**We hope that our answers address the weaknesses of the first version of our paper and that the corrections in the manuscript improved its quality. We are grateful to the great work of the editor that gathered, complied and completed the concerns raised by the reviewers. The resulting summary document was highly constructive and relevant.**

**We remain available to address additional questions if some of our answers were not clear enough.**
